# Patient Characteristics and Changes in Long‐Term Opioid Use Among Patients With Non‐Cancer Pain in Dutch Primary Care 2013–2022: A Population‐Based Cohort Study

**DOI:** 10.1002/ejp.70061

**Published:** 2025-06-28

**Authors:** Loes de Kleijn, Laura Struik, Hanneke J. B. M. Rijkels‐Otters, Alessandro Chiarotto, Bart W. Koes, Jacoline J. van den Driest

**Affiliations:** ^1^ Department of General Practice Erasmus MC, University Medical Center Rotterdam the Netherlands; ^2^ Research Unit of General Practice, Department of Public Health and Center for Muscle and Joint Health University of Southern Denmark Odense Denmark

## Abstract

**Background:**

Pain guidelines recommend de‐prescribing long‐term opioid treatment (LTOT) in non‐cancer pain for reasons of risk–benefit balance. However, the prevalence and changes over time with regard to LTOT in patients with chronic non‐cancer pain in Dutch primary care are unknown. Hence, we examined the prevalence and characteristics and investigated associated diagnoses, comorbidities, co‐medications and changes in prescription numbers between 2013 and 2022.

**Methods:**

This retrospective population‐based cohort study was conducted using the Rijnmond Primary Care Database, which includes over 500,000 patient records from at least 240 GPs within the greater region of Rotterdam. All episodes of LTOT (> 3 months) in patients > 18 years from 2013 to 2022 were included. Descriptive statistics were adopted to characterise the study cohort. The prevalence of LTOT from 2013 to 2022 was calculated per 100 patient years.

**Results:**

Musculoskeletal complaints were the main registered indication by the first prescription of opioids. Patients were more frequently female (66.9%), with a mean age of 62.6 years. Most common comorbidities included diabetes mellitus and depressive disorder. The prevalence of LTOT increased twofold from 0.54% (95% CI: 0.51–0.58) per 100 patient years in 2013 to 1.04% (95% CI: 1.00–1.07) in 2022. The proportion of LTOT episodes solely involving potent opioids slightly increased between 2013 and 2022.

**Conclusions:**

This study demonstrated a twofold increase in the prevalence of LTOT for chronic non‐cancer pain in Dutch primary care from 2013 to 2022. Musculoskeletal pain complaints were the main indication. From 2013 to 2022 potent opioids assumed a more prominent role in LTOT.

**Significance Statement:**

The about twofold increase in long‐term opioid therapy for chronic non‐cancer pain between 2013 and 2022, along with the specific rise in potent opioid prescriptions in Dutch primary care, highlights an urgent need for future studies. These studies should focus on developing strategies to accelerate the implementation of revised primary care pain guidelines, especially given the limited effectiveness of long‐term opioid treatment in non‐cancer pain and the anticipated rise in chronic non‐cancer pain due to Europe's ageing population.

## 1 Introduction

Chronic pain is a leading contributor to the global burden of disease (Blyth et al. 2019). Chronic pain, lasting over 6 months, affects an estimated 12%–34% of Europe's population, with significant regional variations, and is expected to rise due to the ageing population (Todd et al. 2019; Zimmer et al. 2020). In the Netherlands, 25% of people currently suffer from chronic pain (van der Schelde et al. 2024).

The increased recognition of chronic non‐cancer pain, along with aggressive marketing of opioids, contributed, among other factors, to the worldwide increase in opioid prescriptions for pain (Jones et al. 2018). Although, unlike the United States, Europe is not experiencing an opioid epidemic, prescription opioids have risen substantially (Häuser et al. 2021; Kline et al. 2024; Pierce et al. 2021; Pottegård et al. 2023). Moreover, a Dutch study (Kalkman et al. 2019) demonstrated that the rise in opioid prescriptions was accompanied by a concurrent increase in opioid‐related hospital admissions and mortality.

The adverse effects and risks associated with opioid use have raised worldwide concern (Pierce et al. 2021), which is now further intensified by accumulating evidence indicating limited effectiveness in comparison to non‐opioid therapies in terms of risk–benefit balance, particularly in chronic non‐cancer pain treatment (Abdel Shaheed et al. 2016; Chou et al. 2022; Nury et al. 2022). Moreover, due to the addictive nature of opioids, patients are at risk of developing opioid use disorder (American Psychiatric Association 2022). Additionally, growing evidence indicates that patients on long‐term opioid treatment (LTOT) face an increased risk of all‐cause mortality and use of health care utilisation without substantial improvement in quality of life or functioning (Hayes et al. 2018; Tölle et al. 2021).

Consequently, pain guidelines have decreased emphasis on opioid use for managing chronic non‐cancer pain. (Häuser et al. 2017). Nonetheless, de‐prescribing LTOT remains challenging for GPs (de Kleijn et al. 2023; Hamilton et al. 2022). To improve guideline adherence, comprehending the current status of LTOT in primary care is essential. Studies on LTOT prescription patterns in primary care are sparse (Bedson et al. 2016; Black‐Tiong et al. 2021; Foy et al. 2016; Han et al. 2017) and such evidence is currently lacking in the Netherlands (Kalkman et al. 2019; Veldkamp et al. 2024; Weesie et al. 2016). Therefore, this study aims to describe fluctuations in LTOT prescription patterns in primary care in the Rijnmond region of the Netherlands between 2013 and 2022, including types of substances prescribed and explore associated clinical diagnoses, patient characteristics and initiating prescribers. By assessing these aims in a Dutch context, we provide the latest LTOT prevalence data in primary care, insights into the generalizability of prior findings to other Northern European countries and add other valuable insights that could improve pain management and guideline adherence in primary care.

## 2 Methods

### 2.1 Study Population and Settings

This retrospective population‐based cohort study was conducted using the Rijnmond Primary Care Database (RPCD). The RPCD contains over 500,000 pseudonymised electronic patient records of patients registered at approximately 240 GPs in the Rijnmond region, the urban conurbation encompassing the city of Rotterdam in the Netherlands (Schepper 2019). This is a large, predominantly urbanised area with a population of approximately 1.2 million inhabitants, representing 6.7% of the total Dutch population (Central Bureau of Dutch Statistics; Security Region Rotterdam‐Rijnmond). The population is characterised by socioeconomic disadvantages and a plural‐ethnic composition.

The RPCD contains a pseudonymised version of all electronic patient records of participating GP practices. The patient records contain, among others, GP consultation reports, diagnoses based on the International Classification of Primary Care (ICPC) codes, prescriptions issued by GPs, hospital discharge letters and laboratory results. The validity of the RPCD relies on the accuracy and completeness of GPs' registrations.

In the Dutch health care system, GPs are gatekeepers to secondary care. Chronic non‐cancer pain is generally managed by GPs in primary care, but can, in more severe cases, be referred to expert treatment in secondary care in accordance with regulatory guidelines in the Netherlands (Keizer et al. 2023). In the management of non‐cancer pain, GPs are authorised to prescribe opioids, which are dispensed by community pharmacists and reimbursed by the Dutch state for all patients.

### 2.2 Data and Study Outcomes

Patients on LTOT for chronic non‐cancer pain were identified in the RPCD between 1 January 2013 and 31 December 2022. We included patients aged 18 years or older and excluded patients with cancer and patients receiving terminal care 1 year before the first ICPC code or their death. We thereby assumed that all patients with cancer or in terminal care treatment had LTOT prescribed for cancer‐related pain or as part of terminal care. Patients with cancer were identified using ICPC codes referring to cancer diagnosis (Appendix S1). We adhere to LTOT being defined as prescribed opioids for a duration of 90 days or longer in accordance with clinical guidelines (Busse et al. 2017; Dowell et al. 2016) in otherwise heterogenous evidence(de Oliveira Costa et al. 2021; Karmali et al. 2020). To identify LTOT, all opioid prescriptions, isolated and fixed substance combinations were identified using the Anatomical Therapeutic Chemical code ‘N02A’ for opioids (Appendix S2) (WHO Collaborating Centre for Drug Statistics Methodology 2018). Anatomical Therapeutic Chemical codes referring to codeine were excluded, since codeine is no longer reimbursed by the Dutch government since 2013 and is mainly prescribed for non‐pain related symptoms (Matic et al. 2022; Van Luijn 2012). The duration of a LTOT episode was calculated by counting the days from the start of the first opioid prescription until the end of the last prescription in the episode. Opioid prescriptions were considered consecutive when a prescription date followed within 14 days of the end of a previous prescription. Finally, if an opioid prescription would overlap a previous prescription, concurrent opioid use was presumed. Notably, since LTOT episodes were required to last a minimum of 90 days for inclusion, the episodes of LTOT commencing before 2013 or at the end of 2022 could not be included in our analysis, leading to incomplete data collection for 2013 and 2022. In accordance with the current WHO analgesic ladder, opioids in this study were subdivided into ‘weak’ and ‘potent’ opioids (Anekar et al. 2023). ‘Weak’ opioids include tramadol and tramadol combined with non‐opioid analgesics. Remaining opioids were considered ‘potent’ opioids (Anekar et al. 2023).

For all ‘LTOT episodes’, the registered ICPC code coupled to the first opioid prescription was extracted. Moreover, patient characteristics such as gender, age and the area level of social deprivation (SDA) index were extracted. The area level of social deprivation of patients' residences is determined using the SDA index calculated by Devillé and Wiegers (2012) using the following four indicators: the proportion of benefit recipients, the percentage of residents with low income, the density of surrounding addresses and presence of non‐Western foreign residents. Information regarding concurrent drug use and psychiatric comorbidities associated with increased risk for opioid use disorder (Klimas et al. 2019) were examined (Appendix S3). Additionally, comorbidities that are recognised as absolute and relative contraindications to NSAIDs by the Dutch College of General Practitioners (Damen et al. 2022) were examined to provide for a deeper understanding of the cohort's risk profile in relation to non‐opioid pain treatment options (Appendix S3). To explore potential gender and age differences, characteristics will also be presented within gender and age subgroups.

### 2.3 Statistical Analysis

Descriptive statistics were used to describe the study cohort and changes in opioid prescriptions, including prevalence proportions (%) for categorical variables and means (SD) or medians (IQR) for continuous variables. Age was included as a continuous variable. All other descriptive variables, namely living in a socially deprived area (categorised as yes, no or unknown), co‐medications (acetaminophen and other anilides, antidepressants, anxiolytics, gapapentinoids, muscle relaxants, dermal NSAIDs and oral NSAIDs), comorbidities recognised as (relative) contraindications for NSAIDs (acute coronary syndrome, angina pectoris, cerebral vascular accident, chronic kidney disorder, chronic obstructive pulmonary disease, diabetes mellitus, gastric ulcer, heart failure and peptic ulcer) and comorbidities associated with an increased risk of opioid use disorder (affective disorder, attention deficit/hyperkinetic disorder, anxiety disorder, depression, hypochondria, personality disorder, post‐traumatic stress disorder and schizophrenia) were included as categorical variables (Appendix S3).

The daily dose in oral morphine equivalents (OME) in milligrams was calculated using the opioid conversion factors proposed by Nielsen et al. (Nielsen et al. 2016). The prevalence of LTOT was calculated by dividing the number of patients with LTOT occurring within each respective period by 100 patient years. Patient years were defined as the cumulative duration of time that patients contributed to the RPCD (Spronk et al. 2019), thereby accounting for the dynamic nature of the RPCD. In this study, 95% confidence intervals for the prevalence estimates were calculated using the Poisson distribution, which is commonly applied in epidemiological research, particularly when episodes are expressed as rates using person‐time denominators. R Studio was used for calculating the prevalence using the Poisson distribution. To determine the proportion of LTOT for chronic non‐cancer pain being initiated and monitored by a GP, we manually reviewed a random sample of 500 LTOT episodes. From this random sample, we determined the initial prescriber based on consultation registries and hospital letters. The prescriber was classified as ‘unknown’ when the initial or monitoring prescriber could not be identified from the available clinical documentation.

### 2.4 Study Approval

This study was conducted in accordance with the IASP Ethical Guidelines for Pain Research in Humans (Charlton 1995) and was approved by the Board of Directors of the RPCD (Reg. no: 2023‐036). Since we used pseudonymised data, our study was exempt from medical ethical approval under the Dutch Medical Research Act.

## 3 Results

### 3.1 Study Population

A total of 11,994 patients received LTOT between 2013 and 2022, counting 7761 patients on LTOT for chronic non‐cancer pain, contributing to a total of 14,385 episodes of LTOT (Figure 1). An average of 15.2 opioid prescriptions were prescribed per LTOT episode.Of all patients in this cohort 4624 (59.6%) had one episode of LTOT between 2013–2022. Among the 3137 patients having more than one episode, 1577 (50.3%) had two episodes, 712 (22.7%) had three episodes and the remaining 848 (27.0%) had four or more episodes. On average, an episode of LTOT lasted 336 days (range 91–3547 days) and 3683 (25.6%) episodes lasted at least 1 year or more.

**FIGURE 1:**
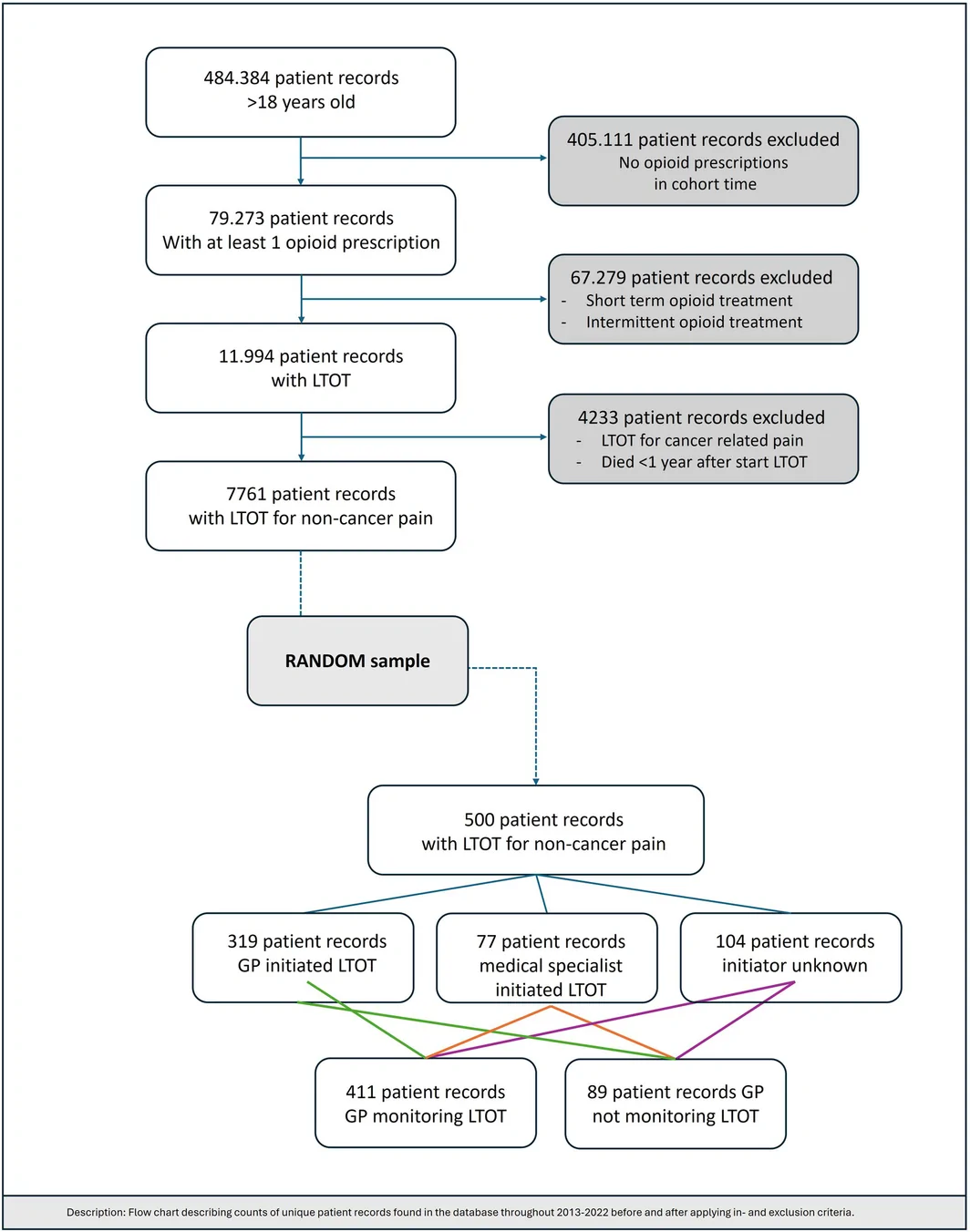
Flowchart of patient records 2013–2022.

### 3.2 Characteristics of Patients With LTOT

The majority of the patients with LTOT in the cohort were female (66.9%) with a mean age of 62.6 years (SD 15.88). Approximately 1874 (23.8%) of patients on LTOT in this cohort had a residency in a deprived area (Table 1). Most common comorbidities found in the medical history of patients on LTOT were depression and diabetes mellitus (Table 1). The most common prescribed concurrent drug use, defined as at least one prescription within an LTOT episode, were antidepressants, acetaminophen and anxiolytics (Table 1). Men on LTOT for non‐cancer pain had a lower mean age at initiation in comparison with women (59.2 vs. 64.1 years) (Table 1). Moreover, diabetes mellitus was more common among men, while peptic disorders were more prevalent among women in the cohort (Table 1). Finally, a higher proportion of younger patients on LTOT resided in socially deprived areas and were more frequently characterised by psychiatric disorders compared to older patients (Table 4 in Appendix S4).

**TABLE 1:** Patient characteristics.

Patient characteristics	Full cohort (*N* = 7761)	Male cohort (*N* = 2570, 33.1%)	Female cohort (*N* = 5191, 66.9%)
Age (years, SD)	62.6 (±15.89)	59.2 (±13.99)	64.1 (±16.44)
Living in a socially deprived area (*N*, %)			
Yes	1847 (23.8%)	716 (27.9%)	1131 (21.8%)
No	5661 (72.9%)	1774 (69.0%)	3887 (74.9%)
Unknown	253 (3.3%)	80 (3.1%)	173 (3.3%)
Comorbidities^a^			
Comorbidities recognised as (relative) contraindications for NSAIDs (*N*, %)			
Acute Coronary Syndrome (K75)	523 (6.7%)	268 (10.4%)	255 (4.9%)
Angina Pectoris (K74)	771 (9.9%)	276 (10.7%)	495 (9.5%)
Cerebral Vascular Accident (K90)	523 (6.7%)	168 (6.5%)	355 (6.8%)
Chronic Kidney Disorder (U99.01)	699 (9.0%)	166 (6.5%)	533 (10.3%)
Chronic Obstructive Pulmonary Disease (R95)	979 (12.6%)	352 (3.7%)	627 (12.1%)
Diabetes Mellitus (T90)	1665 (21.5%)	633 (24.6%)	1032 (19.9%)
Gastric Ulcer (D85, D86)	287 (3.7%)	107 (4.2%)	180 (3.5%)
Heart Failure (K77)	490 (6.3%)	129 (5.0%)	361 (7.0%)
Peptic Disorder (D87)	933 (12.0%)	252 (9.8%)	681 (13.1%)
Comorbidities associated with an increased risk of opioid use disorder (OUD) (*N*, %)			
Affective Disorder (P73)	56 (0.7%)	21 (0.8%)	35 (0.7%)
Attention Deficit/Hyperkinetic Disorder (P21)	102 (1.3%)	41 (1.6%)	61 (1.2%)
Anxiety Disorder (P74)	696 (9.0%)	191 (7.4%)	505 (9.7%)
Depression (P76)	1568 (20.2%)	465 (18.1%)	1103 (21.2%)
Hypochondria (P75)	120 (1.5%)	25 (1.0%)	95 (1.8%)
Personality Disorders (P80)	227 (2.9%)	70 (2.7%)	207 (4.0%)
Post‐Traumatic Stress Disorder (P2.01)	264 (3.4%)	91 (3.5%)	173 (3.3%)
Schizophrenia (P72)	44 (0.6%)	25 (1.0%)	19 (0.4%)
Co‐medication, (*N*, %)^b^			
Acetaminophen and other Anilides (N02BE)	2341 (30.2%)	648 (25.2%)	1693 (32.6%)
Antidepressants (N06A)	2649 (34.1%)	836 (32.5%)	1813 (34.9%)
Anxiolytics, incl. Benzodiazepine (N05BA)	1781 (22.9%)	508 (19.8%)	1273 (24.5%)
Gabapentinoids (N02BF)	1449 (18.7%)	541 (21.1%)	908 (17.5%)
Muscle relaxants (M03)	112 (1.4%)	34 (1.3%)	78 (1.5%)
NSAIDs			
Dermal NSAIDs (M02AA)	264 (3.4%)	70 (2.7%)	194 (3.7%)
Oral NSAIDs (M01AE)	1642 (21.2%)	526 (20.5%)	1116 (21.5%)

^a^ Number of unique patient in this cohort with a co‐morbidity in their medical history at start of their LTOT episode.

^b^ Number of unique patient in this cohort using at least once a co‐medication throughout their LTOT episode(s).

### 3.3 LTOT Episodes for Chronic Non‐Cancer Pain

In 8175 (56.8%) of the episodes of LTOT, the prescription at start was linked to an ICPC code. Of these episodes, approximately 5804 (71.0%) were prescribed for musculoskeletal complaints. The sum of episodes coupled with an ICPC code for low back pain with radiation, low back pain without radiation, back symptoms/complaints, hernia nuclei pulposi, spondylosis of the spine, neck symptoms/complaints and cervical spine syndrome showed that 2737 (33.5%) of LTOT episodes coupled to an ICPC code concerned back and neck pain (Table 2).

**TABLE 2:** Top 20 ICPC codes registered in the first opioid prescription of an episode of LTOT.

ICPC code and diagnosis		*N* (prescriptions)	%
L86.00	Low back pain with radiation	826	10.1%
L03.00	Low back pain without radiation	631	7.7%
L02.00	Back symptoms/complaints	498	6.1%
L86.01	Hernia Nuclea Pulposi (thoracal/lumbal)	424	5.2%
L90.00	Osteoarthritis of the Knee	338	4.1%
L08.00	Shoulder symptoms/complaints	192	2.4%
A01.00	Generalised pain	177	2.2%
L89.00	Osteoarthritis of the Hip	173	2.1%
L20.00	Symptoms of multiple/non‐specified joints	155	1.9%
L91.00	Other osteoarthritis or related disease	154	1.9%
L18.01	Fibromyalgia	152	1.9%
L15.00	Knee symptoms/complaints	149	1.8%
L84.00	Osteoarthritis/Spondylosis of the Spine	135	1.7%
L14.00	Leg/thigh symptoms/complaints	133	1.6%
L01.00	Neck symptoms/complaints	118	1.4%
L13.00	Hip symptoms/complaints	108	1.3%
L83.00	Cervical spine syndrome	105	1.3%
A80.00	Trauma/Injury‐related symptoms	103	1.3%
L95.00	Osteoporosis	103	1.3%
L92.00	Frozen shoulder/Periarthritis humeroscapularis	96	1.2%

*Note:* This table provides an overview the top 20 ICPC codes and diagnosis which were registered at start of the LTOT episode. Notably, in this cohort we could not identify an ICPC code at start of an LTOT episode in 44% of the LTOT episodes.

### 3.4 Prevalence of LTOT and Its Changes Between 2013 and 2022

From 2013 to 2017, the prevalence increased from 0.54 (95% CI: 0.51–0.58) patients on LTOT per 100 patient years in 2013 to 1.00 (95% CI: 0.97–1.05) patients on LTOT per 100 patient years in 2017 (Figure 2). From 2018 up to 2022, the prevalence appears stable, fluctuating around 1.07 (95% CI: 1.02–1.11) to 1.04 (95% CI: 1.00–1.07) per 100 patient years. The prevalence calculated over 2013–2022 is 0.99 (95% CI: 0.98–1.00) per 100 patient years.

**FIGURE 2:**
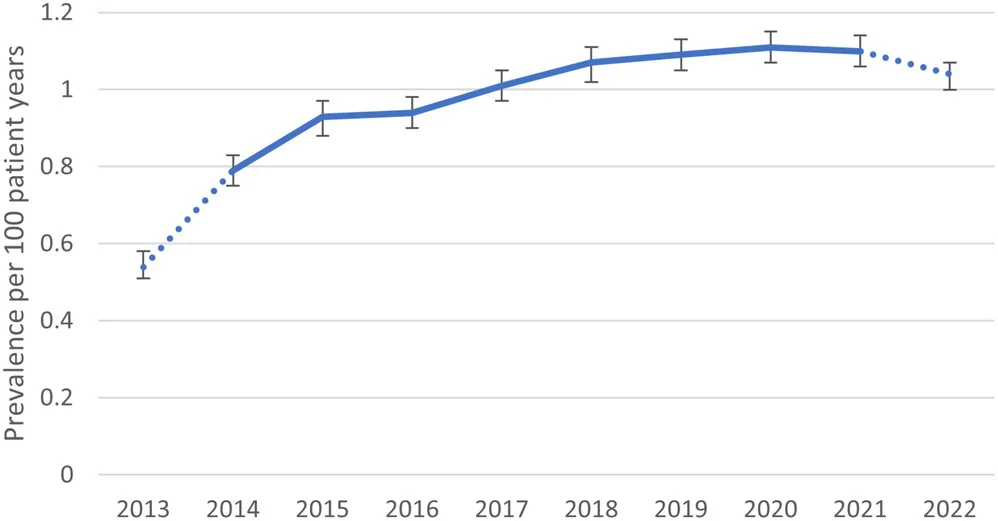
Prevalence of LTOT 2013–2022.

### 3.5 Changes in Opioid Types Prescribed for LTOT Between 2013 and 2022

In 47.8% of LTOT episodes, patients received solely weak opioids and in 37.7% of episodes, solely potent opioids. The longer an episode, the more likely it is to receive a potent opioid (Table 3). 59.8% of all opioid prescriptions in this cohort consist of ‘potent’ opioids. LTOT prescriptions increasingly included potent opioids between 2013 and 2022, mainly driven by an increase in LTOT prescriptions of oxycodone and fentanyl among patients with LTOT for chronic non‐cancer pain (Table 3 and Figure 3). In 2013, oxycodone accounted for 49.2% of potent opioid prescriptions, rising to 55.9% by 2022. Meanwhile, fentanyl's share increased from 21.4% in 2013 to 28.1% in 2022. During the same period, buprenorphine prescriptions declined from 21.0% to 7.7% of all potent opioid prescriptions. Likewise, morphine prescriptions declined from 8.3% to 6.0% of all potent prescriptions for LTOT between 2013 and 2022.

**TABLE 3:** Type of opioid prescriptions across five different LTOT episode durations.

LTOT episode duration	Number of episodes	Proportion LTOT episodes consisting of weak opioids	Proportion LTOT episodes consisting of weak and potent opioids	Proportion LTOT episodes consisting of potent opioids
3–6 months	6607	3483 (53%)	827 (12%)	2297 (35%)
6–9 months	2819	1321 (47%)	379 (13%)	1119 (40%)
9–12 months	1276	599 (47%)	179 (14%)	498 (39%)
1–2 years	2190	935 (43%)	328 (15%)	927 (42%)
> 2 years	1493	535 (36%)	379 (25%)	579 (39%)

*Note:* Number of LTOT episodes (%) consisting of prescriptions consisting of weak, potent or a combination in five LTOT episode duration categories.

**FIGURE 3:**
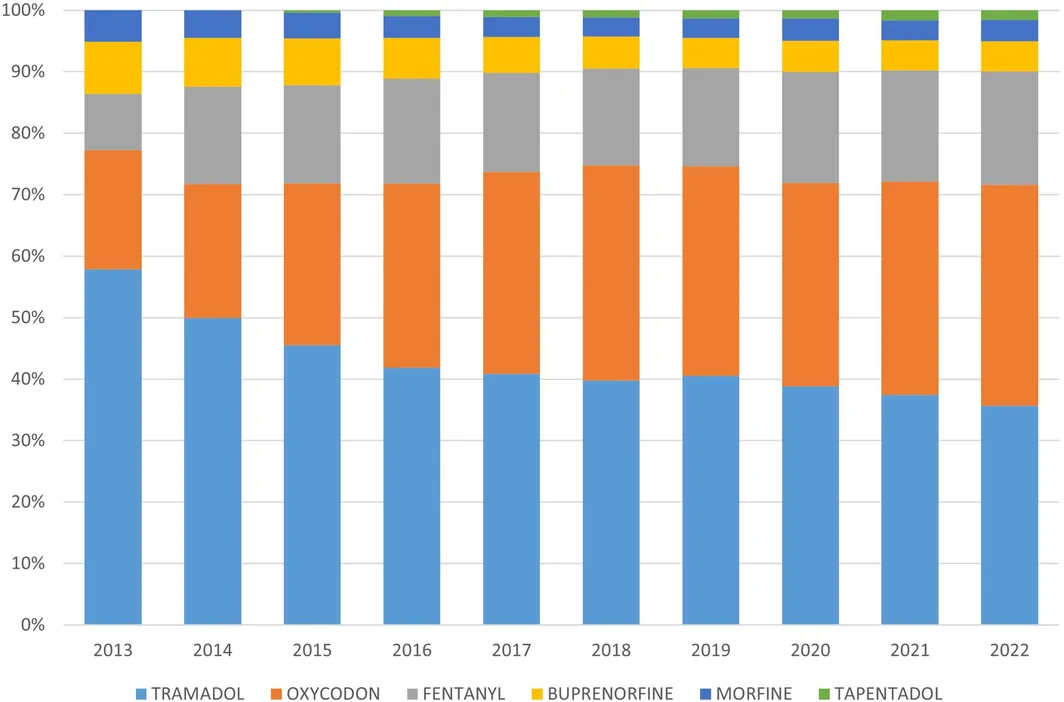
Barplot displaying opioid substances prescribed as proportions of all opioids prescribed in LTOT episodes between 2013 and 2022.

The median (IQR) oral morphine equivalent daily dose over 2013–2022 was 28.9 mg (19.3–46.9 mg). The oral morphine equivalent daily dose increased from median of 27.1 mg/day in 2013 to 28.1 mg/day in 2022 (Figure 4). Notably, the daily dose increased mainly in episodes of LTOT with prescriptions of only potent opioids, whereas the daily dose remained stable in episodes with only weak opioid prescriptions or combined weak and potent opioid prescriptions (Figure 4).

**FIGURE 4:**
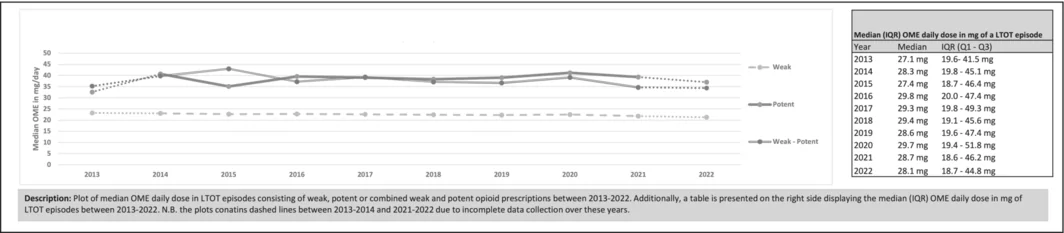
Changes in median oral morphine equivalent daily dose.

Between 2013 and 2022, LTOT episodes comprising only potent opioid prescriptions steadily increased, with a simultaneous decrease in LTOT episodes comprising only weak opioid prescriptions (Figure 5).

**FIGURE 5:**
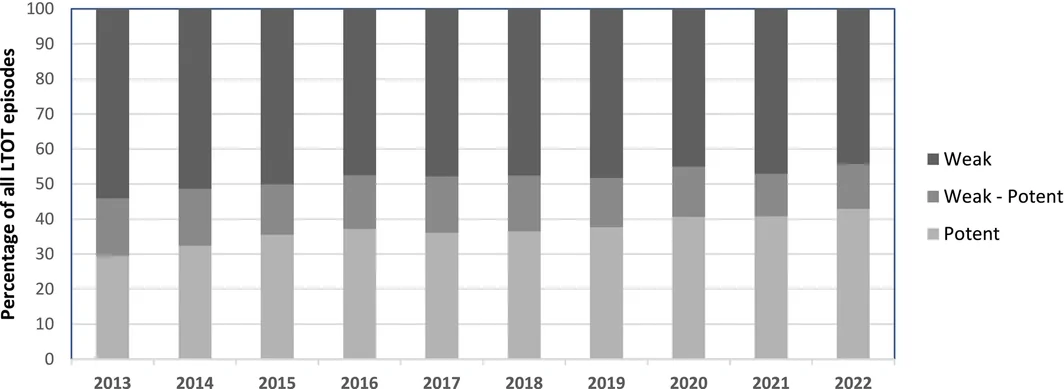
Weak and potent opioids in LTOT episodes 2013–2022.

In 11.8% of LTOT episodes involving only potent opioids, there was a preceding episode of weak opioids or a combination of weak and potent opioids. Notably, the number of prescriptions per LTOT episode also increased from 2013 to 2022 from an average of 6.6–10.4 prescriptions per episode. At the same time, the mean length of the prescriptions remained stable.

### 3.6 LTOT Initiation and Monitoring

Based on the random sample of 500 random patients with LTOT between 2013 and 2022, LTOT was initiated by the GP in 319 (63.8%) patients, by a medical specialists in secondary care in 77 (15.4%) patients and in 104 (20.8%) patients the initial prescriber could not be identified (Figure 1). Additionally, 411 (82.2%) patients in this random sample were monitored within primary care with the GP as the principal clinician (Figure 1).

## 4 Discussion

Between 2013 and 2022, 14.385 episodes of LTOT for chronic non‐cancer pain were identified in the RPCD. Between 2013 and 2022, the majority of LTOT for chronic non‐cancer pain was prescribed for musculoskeletal pain complaints. The prevalence of LTOT in the RPCD increased nearly twofold from 0.54 (95% CI: 0.51–0.58) patients on LTOT per 100 patient years in 2013 to 1.04% (95% CI: 1.00–1.07) patients on LTOT per 100 patient years in 2022. Prescriptions of potent opioids increased, whereas prescriptions for weak opioids decreased. Furthermore, there was an increase in the proportion of LTOT episodes solely involving potent opioids.

Although it is challenging to compare changes in opioid prescribing across countries due to differences in prescribing policy and legislation, a rise in potent opioids seems a persistent outcome across high‐income countries (Bedson et al. 2019, 2016; Black‐Tiong et al. 2021; Foy et al. 2016; Han et al. 2017; Scherrer et al. 2022). In Dutch primary care, this increase of potent opioids, especially oxycodone and fentanyl, for non‐cancer pain has been confirmed by various studies (Schepens et al. 2019; Veldkamp et al. 2024; Weesie et al. 2016). Our study further contributes by indicating not only an increase in LTOT for chronic non‐cancer pain, but more specifically, an increase in LTOT consisting of potent opioids, particularly oxycodone and fentanyl.

Oral morphine has been the preferred potent opioid in Dutch primary care until 2023. The specific rise in oxycodone and fentanyl between 2013 and 2022 found in our study is noteworthy, as increasing evidence has revealed an increased abuse potential in oxycodone compared to other opioid substance types (Kibaly et al. 2021; Remillard et al. 2019). The specific increase of LTOT comprising only potent opioids and a successive decrease of LTOT episodes comprising only weak opioids aligns with findings of a similar study performed in the UK (Bedson et al. 2016). These findings are at odds with primary care guidelines over the past decade in both the Netherlands and the United Kingdom, which have continued to recommend doctors to concur with the WHO analgesic ladder, initiating weak opioids before initiating potent opioids in non‐cancer pain (Anekar et al. 2023; Damen et al. 2022; Federatie Medisch Specialisten 2023). Additionally, they are incompatible with increasing studies indicating adverse health outcomes and increased mortality risk in patients on potent opioids compared to weak opioids (Acharya et al. 2023; Chou et al. 2014; Dunn et al. 2019; Expert Committee on Drug Dependence 2018; Vosburg et al. 2018).

The observed decline in the slope or potential stabilisation of the prevalence of LTOT for chronic non‐cancer pain between 2019 and 2022 may suggest a decline in the incidence of LTOT. This finding is aligned with British and Australian studies reporting declining incidence rates of LTOT episodes in 2011 and 2017, respectively (Bedson et al. 2016; Black‐Tiong et al. 2021). Future research should explore whether these changes persist and whether they are preliminary effects of healthcare initiatives and changes in guideline recommendations, or, as Bedson et al. (2016) explained, a ‘spill‐over effect’ from stricter prescriber policies in the United States. Exploring future trends of LTOT for chronic non‐cancer pain in primary care are even more necessary, considering that most primary care pain guidelines have only recently begun to place less emphasis on opioids (Damen et al. 2022; National Institute for Health and Care Excellence 2021). Consequently, the impact of these recent guideline changes on LTOT prevalence remains unclear, particularly considering the lagged time effects of guideline changes on clinical practice (Morris et al. 2011). The simultaneous increase in chronic non‐cancer pain prevalence due to the ageing European population (Zimmer et al. 2020) and increasing evidence of opioids limited effectiveness in acute musculoskeletal pain complaints, such as back and neck pain (Jones et al. 2023; Krebs et al. 2018; Licciardone et al. 2024; Nury et al. 2022), necessitates an urgent need for studies examining underlying reasons for adherence and non‐compliance of pain guidelines in primary care. In fact, it necessitates the need to explore additional strategies to accelerate the implementation of revised guidelines within primary care.

Similar retrospective studies on LTOT have been conducted in Australia, Britain and the United States (Bedson et al. 2019; Black‐Tiong et al. 2021; Foy et al. 2016; Han et al. 2017; Jani et al. 2020; Scherrer et al. 2022). Our results align with most of these previous findings, suggesting that our results are generalisable to other high‐income countries. Previous studies focused on describing LTOT among specific subgroups (Han et al. 2017) or pain complaints (Bedson et al. 2019, 2016; Black‐Tiong et al. 2021) or investigated opioid prescription trends (Jani et al. 2020). Our study, however, specifically provided insight into both the characteristics of patients on LTOT for chronic non‐cancer pain and opioid prescription changes. Finally, unlike other register‐based studies that often define LTOT as 3 prescriptions per 90 days, we applied a stricter definition, requiring prescriptions to be issued within 14 days of each other. Hereby, we reduced the likelihood of erroneous inclusion of intermittent and short‐term opioid users in our cohort. Additionally, the implementation of this stricter definition probably explains the higher LTOT prevalences found in previous studies (Bedson et al. 2016; Black‐Tiong et al. 2021).

Since the initial prescriber could not be ascertained without a search within each individual patient record, the changes delineated by this research cannot be solely attributed to the prescribing practices of GPs. Instead, the observed changes reflect patterns in LTOT episodes in a primary care setting, involving various medical specialties. However, with 63.8% of the LTOT in our random sample being initiated by a GP, it seems that GPs play a pivotal role in prescribing opioids among patients with chronic non‐cancer pain. This aligns with a previous opioid study performed in Denmark (Pottegård et al. 2023) and is supported by a recent report by the National Taskforce on appropriate opioid prescribing that demonstrated GPs and medical specialists equally initiate LTOT episodes (Coolen van Brakel 2021).

Like any other database study, registration in patient records can contain omissions and mistakes. This inherent limitation of database studies, however, does not undermine the internal validity of study results. With ICPC codes not being mandated for reimbursement of prescriptions in the Netherlands, we believe that the 44% of the LTOT episodes not linked with an ICPC code at start are caused by random register omissions and do not undermine the results of our study.

Given that our data were derived from a predominantly urbanised area characterised by socioeconomic disadvantages, it is important to acknowledge the limitations regarding the generalisability of our findings to the rest of the Netherlands. Given the absence of data regarding the economic or ethnic backgrounds of the included patients, it was not possible to assess their potential impact on our results. However, the results of Veldkamp et al. (2024) demonstrating increased opioid prescriptions for non‐cancer pain between 2013 and 2022 in a predominantly rural area of the Netherlands and the results of national pharmacist studies (Lambooij et al. 2022, 2024) demonstrating similar changes between 2020 and 2023, collectively support our results, making them indicative of a national tendency.

We could not assess if the increase in LTOT episodes during 2013–2022 was attributable to a parallel rise in the prevalence of chronic non‐cancer pain in the Netherlands for a lack of data. Nonetheless, any increase in LTOT episodes over time warrants attention given the limited efficacy in pain relief and the risks of severe side effects from opioids in the treatment of chronic non‐cancer pain (Chou et al. 2020; Nury et al. 2022).

As the RPCD is a dynamic cohort, we could not differentiate initial opioid users from previous opioid users from the patient records at time of patient entry into the register. We anticipate that these unrecorded effects would likely underestimate the length of LTOT episodes in current data and may have obscured differences in patient characteristics between patients on extensive LTOT and patients on LTOT for shorter instances. The steep increase between 2013 and 2014 and the negative slope between 2021 and 2022 are probably partially explained by incomplete data collection in the years 2013 and 2022. These periods should therefore be interpreted with caution, as we do not find these lower prevalences of LTOT episodes likely based on our longitudinal data.

## 5 Conclusions

This study found an about twofold increase in the prevalence of LTOT episodes for chronic non‐cancer pain between 2013 and 2022, stabilising from 2019. Potent opioids were more often prescribed in LTOT episodes with a particular increase in the prescription of oxycodone and fentanyl. Musculoskeletal pain complaints were more prevalent than other diseases as a main indication for LTOT episodes in patients with chronic non‐cancer pain. With an expected increase in chronic non‐cancer pain prevalence and continuing increase of evidence demonstrating limited effectiveness of LTOT in patients with chronic non‐cancer pain, future studies need to determine whether the stabilisation of LTOT prevalence observed in this study serves as an early indicator of positive progress. Meanwhile, future studies should also examine underlying reasons for adherence and non‐compliance with primary care pain guidelines and explore additional strategies to accelerate the implementation of the revised pain guidelines in order to close the gap between research‐based knowledge and clinical practice.

## Author Contributions

All the authors are listed and contributed substantially. This study was designed by L.K., L.S., A.C., H.J.B.M.R.‐O., B.W.K. and J.J.D. The analysis was performed by L.K. and L.S. under the supervision of J.J.D., and the results were critically examined by all authors. L.K. had a primary role in preparing the manuscript, which was edited by all authors equally. All authors have approved the final version of the manuscript and agree to be accountable for all aspects of the work.

## Conflicts of Interest

The authors declare no conflicts of interest.

## Supporting information


**Appendix S1.** Overview of ICPC codes of malignancy diagnosis excluded from the cohort.
**Appendix S2.** All Opioid types included in this study according to the ATC code N02A.
**Appendix S3.** Overview of ATC‐codes and ICPC codes of investigated concurrent drug use and comorbidities.
**Appendix S4.** Table 4 demonstrating patient characteristics in gendered age cohorts.
