# Supplementary material for: Patient Characteristics and Changes in Long‐Term Opioid Use Among Patients With Non‐Cancer Pain in Dutch Primary Care 2013–2022: A Population‐Based Cohort Study
Source: Eur J Pain. 2025 Jun 28;29(6):e70061. doi: 10.1002/ejp.70061 (PMC13733643; doi:10.1002/ejp.70061)
Supplement: Supplementary file 1 [file EJP-29-0-s001.docx]

**Legend of tables, figures and appendices:**

Table 1 Patient characteristics

Table 2: Top 20 ICPC codes registered in the first opioid prescription of an episode of LTOT.

Table 3: Type of Opioid prescriptions across Five different LTOT episode durations

Figure 1: Figure 1 flowchart of patient records 2013-2022

Figure 2: Prevalence of LTOT 2013-2022

Figure 3: Barplot displaying opioid substances prescribed as proportions of all opioids prescribed in LTOT episodes between 2013-2022

Figure 4: Changes in median Oral Morphine Equivalent daily dose

Figure 5: Weak and potent opioids in LTOT episodes 2013-2022

Appendix S1: Overview ICPC codes of cancer diagnosis excluded from the cohort

Appendix S2: All Opioid types included in this study according to the ATC code N02A

Appendix S3: Overview of ATC-codes and ICPC codes of investigated concurrent drug use and comorbidities.

Appendix S4: Table 4 demonstrating patient characteristics in gendered age cohorts.

**Appendix S1**

**Overview ICPC codes of cancer diagnosis excluded from the cohort**

**ICPC code	Diagnosis**

A79	Malignancy NOS

B72	Hodgkin’s disease / lymphoma

B72.01	Hodgkin’s disease/ lymphoma

B72.02	Non-Hodgkin’s lymfoma

B73	Leukaemia

B74	Malignant neoplasm blood other

B74.01	Multiple myeloma

D74	Malignant neoplasm stomach

D75	Malignant neoplasm colon/ rectum

D76	Malignant neoplasm pancreas

D77	Malignant neoplasm digestive other/ NOS

F74	Neoplasm of the eye/ adnexa

H75	Neoplasm of the ear

K72	Neoplasm of the cardiovascular system

L71	Neoplasm of the musculoskeletal system

L71.01	Malignant neoplasm musculoskeletal

N74	Malignant neoplasm the nervous system

N76	Neoplasm nervous system unspec.

R84	Malignant neoplasm bronchus/ lung

R85	Malignant neoplasm respiratory, other

S77	Malignant neoplasm of skin

S77.03	Melanoma

T71	Malignant neoplasm thyroid

T73	Neoplasm endocrine oth/ unspecified

U75	Malignant neoplasm of kidney

U76	Malignant neoplasm of bladder

U77	Malignant neoplasm urinary tract

U79	Neoplasm urinary tract NOS

W72	Malignant neoplasm related to pregnancy

X75	Malignant neoplasm cervix

X76	Malignant neoplasm breast female

X77	Malignant neoplasm genital other (f)

X77.01	Endometrial carcinoma

X77.02	Ovarian cancer

X81	Genital neoplasm other/ unspecified (f)

Y77	Malignant neoplasm prostate

Y78	Malignant neoplasm male genital other

**Appendix S2**

**All Opioid types included in this study according to the ATC code N02A**

See ATC/DDD Index 2024 of the WHO Collaborating Centre for Drugs Statistics Methodology.

See <https://atcddd.fhi.no/atc_ddd_index/> . We excluded all codeine drug types, see methods.

**N02AA Natural opium alkaloids**

N02AA01 Morphine

N02AA02 Opium

N02AA03 Hydromorphone

N02AA04 Nicomorphine

N02AA05 Oxycodone

N02AA10 Papaveretum

N02AA11 Oxymorphone

N02AA51 Morphine, combinations

N02AA53 Hydromorphone and naloxone

N02AA55 Oxycodone and naloxone

N02AA56 Oxycodone and naltrexone

**N02AB Phenylpiperidine derivatives**

N02AB01 Ketobemidone

N02AB02 Pethidine

N02AB03 Fentanyl

N02AB52 Pethidine, combinations excluding psycholeptics

QN02AB53 Fentanyl, combinations excluding psycholeptics

N02AB72 Pethidine, combinations with psycholeptics

QN02AB73 Fentanyl, combinations with psycholeptics

**N02AC Diphenylpropylamine derivatives**

N02AC01 Dextromoramide

N02AC03 Piritramide

N02AC04 Dextropropoxyphene

N02AC05 Bezitramide

N02AC52 Methadone, combinations excluding psycholeptics

N02AC54 Dextropropoxyphene, combinations excluding psycholeptics

N02AC74 Dextropropoxyphene, combinations with psycholeptics

QN02AC90 Methadone

**N02AD Benzomorphan derivatives**

N02AD01 Pentazocine

N02AD02 Phenazocine

N02AD51 Pentazocine and naloxone

**N02AE Oripavine derivatives**

N02AE01 Buprenorphine

QN02AE90 Etorphine

QN02AE99 Oripavine derivatives, combination

**N02AF Morphinan derivatives**

N02AF01 Butorphanol

N02AF02 Nalbuphine

**N02AJ Opioids in combination with non-opioid analgesics**

N02AJ13 Tramadol and paracetamol

N02AJ14 Tramadol and dexketoprofen

N02AJ15 Tramadol and other non-opioid analgesics

N02AJ16 Tramadol and celecoxib

N02AJ17 Oxycodone and paracetamol

N02AJ18 Oxycodone and acetylsalicylic acid

N02AJ19 Oxycodone and ibuprofen

N02AJ22 Hydrocodone and paracetamol

N02AJ23 Hydrocodone and ibuprofen

**N02AX Other opioids**

N02AX01 Tilidine

N02AX02 Tramadol

N02AX03 Dezocine

N02AX05 Meptazinol

N02AX06 Tapentadol

N02AX07 Oliceridine

N02AX51 Tilidine and naloxone

**Appendix S3**

**Overview of ATC-codes and ICPC codes of investigated concurrent drug use and comorbidities.**

**ATC code	Drug types**

N02BE	Analides, such as paracetamol

M02AA	NSAID, dermal

M01AE	Propionic acid derivatives, i.e. NSAIDs

NO2BF	Gabapentinoids

M03	Muscle relaxants

N05BA	anxiolytics, including benzodiazepine

N06A	antidepressants

**ICPC code	Diagnosis**

T90	Diabetes mellitus

K77	Heart failure

K74	Angina pectoris

K75	Acute coronary syndrome

K90	Cerebral vascular accident

U99.01	chronic kidney disorder

R95	chronic obstructive pulmonary disease (COPD)

D85/D86	Gastric ulcer

D87	Peptic ulcer

P2.01	post-traumatic stress disorder (PTSD)

P72	Schizofrenia

P74	anxiety disorder

P75	hypochondria

P76	Depression

P80	Personality disorder

| **Table 4: Patient characteristics in gendered age cohorts** | | | | | |
| --- | --- | --- | --- | --- | --- |
| **Patient characteristics** |  | **Male 18-65 years**  **(N=1671, 21.5%)** | **Male >65 years**  **(N=899, 11.6%)** | **Female 18-65 years**  **(N=2561, 33.0%)** | **Female >65 years**  **(N 2630, 33.9%)** |
| Living in a socially deprived area (N,%): |  |  |  |  |  |
|  | Yes | 541 (32.4%) | 175 (19.5%) | 690 (26.9%) | 441 (16.8%) |
|  | No | 1073 (64.2%) | 701 (78 %) | 1773 (69.2%) | 2114 (80.4%) |
|  | Unknown | 57 (3.4%) | 23 (2.6%) | 98 (3.8%) | 75 (2.9%) |
| Co-morbidities** |  |  |  |  |  |
| Comorbidities recognized as (relative) contraindications for NSAIDs (N,%) |  |  |  |  |  |
|  | Diabetes mellitus (T90) | 328 (N=19.6%) | 305 (33.9%) | 348 (13.6%) | 684 (26%) |
|  | Heart failure (K77), | 27 (1.6%) | 102 (11.3%) | 40 (1.6%) | 321 (12.2%) |
|  | Angina pectoris (K74) | 113 (6.8%) | 163 (18.1%) | 98 (3.8%) | 397 (15.1%) |
|  | Acute coronary syndrome (K75) | 109 (6.5%) | 159 (17.7%) | 58 (2.3%) | 197 (7.5%) |
|  | Cerebral vascular accident (K90) | 68 (4.1%) | 100 (11.1%) | 99 (3.9%) | 256 (9.7%) |
|  | Chronic kidney disorder (U99.01) | 36 (2.2%) | 130 (14.5%) | 81 (3.2%) | 452 (17.2%) |
|  | Chronic Obstructive Pulmonary Disease (COPD) (R95) | 153 (9.2%) | 199 (22.1%) | 232 (9.1%) | 395 (15.0%) |
|  | Gastric ulcer (D85, D86) | 63 (3.8%) | 44 (4.9%) | 77 (3.0%) | 103 (3.9%) |
|  | Peptic disorder (D87) | 159 (9.5%) | 93(10.3%) | 365 (14.3%) | 316 (12.0%) |
| Comorbidities associated with an increased risk of opioid use disorder (OUD) (N,%) |  |  |  |  |  |
|  | Post-traumatic stress disorder (P2.01) | 80 (4.8%) | 11 (1.2%) | 155 (6.1%) | 18 (0.7%) |
|  | Schizophrenia (P72) | 22 (1.3%) | 3 (0.3%) | 12 (0.5%) | 7 (0.3%) |
|  | Anxiety disorder (P74) | 153 (9.2%) | 38 (4.2%) | 323 (12.6%) | 182 (6.9%) |
|  | Hypochondria (P75) | 19 (1.1%) | 6 (0.7%) | 65 (2.5%) | 30 (1.1%) |
|  | Depression (P76) | 344 (20.6%) | 121 (13.5%) | 662 (25.8%) | 441 (16.8%) |
|  | Personality disorders (P80) | 65 (3.9%) | 5 (0.6%) | 185 (7.2%) | 22 (0.8%) |
|  | Affective disorder (P73) | 18 (1.1%) | 3 (0.3%) | 25 (1.0%) | 10 (0.4%) |
|  | Attention deficit/hyperkinetic disorder (P21) | 38 (2.3%) | 3 (0.3%) | 59 (2.3%) | 2 (0.1%) |
| Description:  * Number of unique patient in this cohort with a co-morbidity in their medical history at start of their LTOT-episode. | | | | | |

**Appendix S4**
